# EDP-mitotane in children: reassuring evidence of reversible side-effects and neurotoxicity

**DOI:** 10.1007/s12672-022-00486-1

**Published:** 2022-04-18

**Authors:** Rebecca V. Steenaard, Marieke Rutjens, Madeleine H. T. Ettaieb, Max M. van Noesel, Harm R. Haak

**Affiliations:** 1 Department of Internal Medicine, Máxima MC, Ds. Th. Fliedernerstraat 1, Eindhoven/Veldhoven, 5631 BM The Netherlands; 2 CAPHRI School for Public Health and Primary Care, Ageing and Long-Term Care, Maastricht University, Maastricht, The Netherlands; 3 Department of Internal Medicine, Tergooi, Hilversum/Blaricum, The Netherlands; 4 Princes Máxima Center for Pediatric Oncology, Utrecht, The Netherlands; 5 Division of Cancer and Imaging, University Medical Centre Utrecht, Utrecht, The Netherlands; 6 Department of Internal Medicine, Maastricht University Medical Centre+, Maastricht, The Netherlands

**Keywords:** Adrenocortical carcinoma, Childhood, Mitotane, Chemotherapy, Side-effects, Neurotoxicity

## Abstract

Adrenocortical carcinoma affects one in 5 million children each year. Since prognosis for children older than 4 years is limited, clinicians often choose aggressive treatment with etoposide, doxorubicin, cisplatin (EDP) and mitotane after resection. However, little is known about the impact of EDP-mitotane in children. We provide an overview of case-reports and case series listing side-effects and neurotoxicity of EDP-mitotane in children. Fourteen studies were identified describing a range of gastro-intestinal, endocrine, developmental and neuropsychological side-effects. Neurotoxicity included motor- and speech delay, decreased concentration and lower school performance. These side-effects appear to be reversible after mitotane discontinuation. We have added our own experience with a 10 year old girl with advanced adrenocortical carcinoma treated with EDP and 2 years of mitotane after irradical resection. She developed an impactful, but reversible, decrease in cognitive development measured by a standardized neuropsychological assessment before, during and after mitotane therapy. This decrease was mostly measurable in terms of decreased processing speed and concentration and a significant drop in school performance. Combined with fatigue and insecurity, this caused problems in short-term memory and the need to change her school type. In conclusion, EDP-mitotane is associated with several side-effects including neurotoxicity in pediatric cases, all reversible after mitotane discontinuation.

## Introduction

One in 5 million children is affected by adrenocortical carcinoma (ACC) each year Worldwide [1]. The majority is younger than 4 years of age at the time of the diagnosis. Children most often present with signs of virilization (> 80%), and less often with signs of Cushing or Conn syndrome. Abdominal pain is also reported as presenting symptom [2].

The pathogenesis appears to be different in children than in adults. TP53 mutations are more common in children compared to adults, both sporadic and as germline Li-Fraumeni syndrome [3]. Mutations in this gene are very common in the South of Brazil resulting in an incidence of 3–4 per million children [4]. Other syndromes related to childhood ACC are Beckwith-Wiedemann syndrome (11p15 imprinting), Lynch syndrome (mismatch repair genes), multiple endocrine neoplasia type 1 (MEN1), familial adenomatous polyposis (APC), neurofibromatosis type 1 (NF1) and Carney complex (2p16 and 17q22-24) [5, 6]. In children without germline mutations, somatic mutations in these chromosomal regions have also been discovered. From these syndromes different pathways have been identified in relation to ACC pathogenesis in both children and adults, such as p53/Rb signaling, insulin-like growth factor, β-catenin pathway and chromatin remodeling [7]. Differences between child and adult pathogenesis have been identified in pathways related to development of the adrenal cortex (DAX1/SF1/Sonic hedgehog) [8]. The clinical consequences of these differences have not yet been clarified.

In children the prognosis for survival is known to be better than in adults. The 5-year survival of children up to the age of 4 years is 91%, compared to 30% in older children [1, 2, 9]. Factors influencing prognosis are age, tumor size, distant metastases, endocrine overproduction and Ki-67 index [1, 9, 10]. The majority of children younger than 4 years present with localized (70%) and small tumors (90% <10 cm), which explains their favorable prognosis. However, the prognosis in advanced ACC is unfavorable in children as well. Additional treatment with etoposide, doxorubicin, cisplatin (EDP) and mitotane is therefore advised, even though there is no exclusive evidence that treatment with mitotane or EDP gives a survival advantage in children [2, 9, 11–14].

Mitotane is the cornerstone in treating aggressive forms of ACC in both children and adults. This drug is a metabolite of the insecticide DDT (dichlorodiphenyltrichloroethane) and is known for its high toxicity. Monitoring of mitotane plasma levels has been shown to be very important, as kinetic properties are widely variable between patients. The therapeutic lower limit is defined as 14 mg/L, the toxic upper limit is set on 20 mg/L [15–17]. The most common side-effects are complaints of nausea and diarrhea. Neurotoxic side-effects can also occur, which often present as an attention deficit, disturbed sense of balance or memory dysfunction [15]. The side-effects of EDP are well described in children and include hearing loss, pancytopenia and neutropenic fever. The side-effects of mitotane on the other hand, are mostly based on studies in adults [18]. We do know that mitotane is often discontinued due to intolerance in both children and adults [19, 20]. Little is known though about the impact of mitotane on a developing child and especially on cognitive development. We therefore aimed to provide an overview of reported side-effects and neurotoxicity of EDP-mitotane in children with ACC. We have added our own experience with a 10 year old girl who developed an impactful, but reversible, delay in cognitive development during mitotane treatment.

## Materials and methods

We performed a literature search into case reports and case series describing side-effects and neurotoxicity in children with ACC or Cushing treated with EDP and/or mitotane. We searched Medline for articles published in English until January 2021. We used the following keywords: adrenocortical carcinoma, adrenocortical neoplasm, Cushing syndrome, chemotherapy, mitotane, child or pediatric.

We included case reports and case series that described side-effects, neurotoxicity or cognitive development in pediatric patients with ACC or Cushing syndrome treated with either mitotane and/or EDP or specific components of EDP. Studies were excluded if the participants were older than 18 years of age. The search, exclusion and data extraction procedures were performed by two authors independently, and discrepancies were resolved through discussion. A total of 989 articles were identified by the search. After full-text review, we included 14 studies, summarized in Table 1. We have compared the results from these studies to reported side-effects and neurotoxicity in adults during EDP-mitotane therapy.

We have added our own experience with a 10 year old girl with advanced ACC treated with EDP-mitotane after irradical resection. All data within this case report are presented in consultation and with the explicit consent of the patient and her parents. The study has been reviewed by the local Ethical Committee.

## Case report

A Caucasian 10-year-old girl presented with abdominal pain, unexplained weight gain, typical cushingoid appearance, severe acne and excessive hair growth on the arms, legs and pubic area (Tanner B1, P3). Imaging showed a 12 cm right sided adrenal tumor with no distant metastases. During surgery, tumor depositions on the peritoneum and around the tumor were seen. Pathology was consistent with ACC, as defined in the Wieneke criteria, with a KI-67 proliferation index of 10% [21]. The resection was determined as irradical with microscopic residues (R1). Genetic testing showed no germline mutations.

**Fig. 1:**
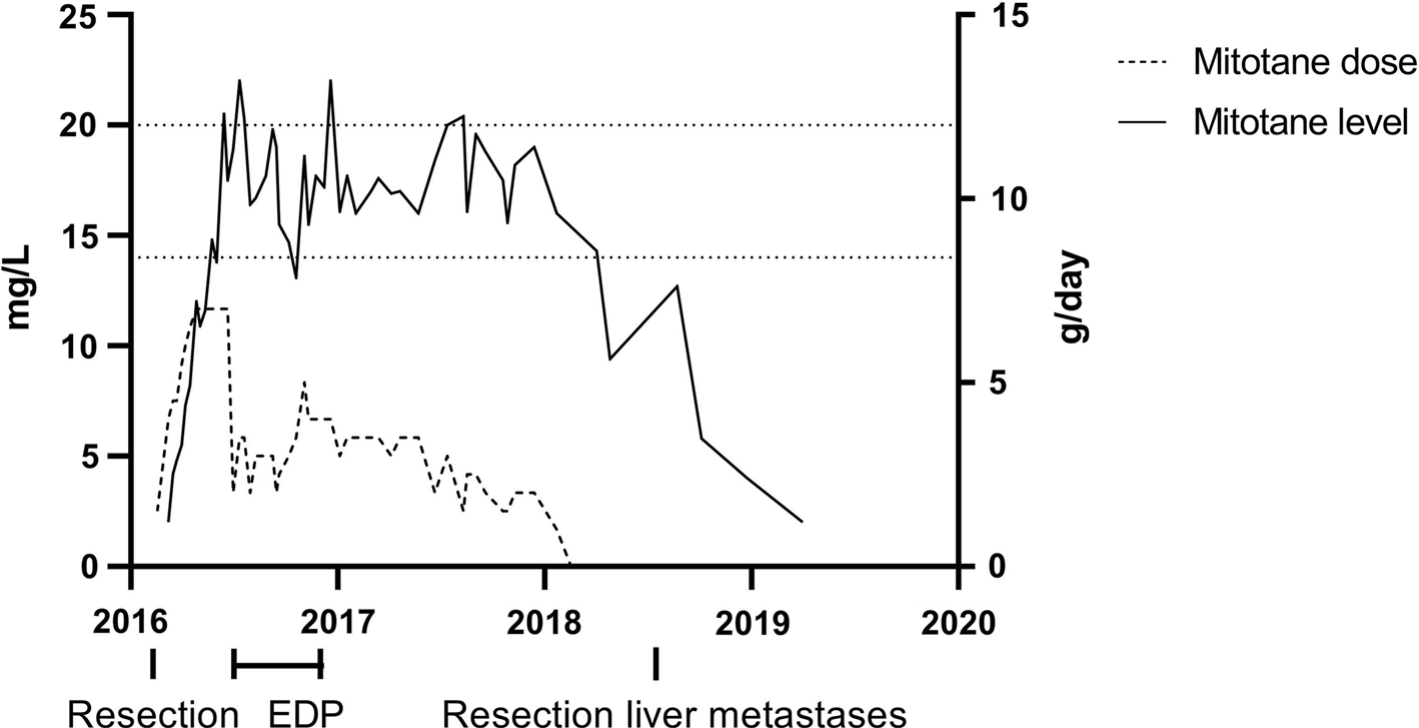
Timeline with treatments, mitotane level (mg/L) and mitotane dose (g/day). *EDP* etoposide, doxorubicin, cisplatin in eight cycles

She completed 8 cycles of adjuvant EPD and mitotane, with hydrocortisone and fludrocortisone supplementation. In total, she received 2 years of mitotane treatment at adequate plasma levels with an average dose of 3 g per day (Fig. 1). Six months after completion of treatment, she developed a liver metastasis of 1.1 cm, which was completely resected through wedge excision. No additional treatment was started afterward. In the three years since, no new lesions have been detected.

During both EDP and mitotane treatment she experienced nausea, which was controlled with various antiemetics. She developed central hypothyroidism and hypocalcemia, requiring levothyroxine, calcium and vitamin D. She experienced well known side-effects of EDP such as neutropenia, anemia and mouth sores. Emotionally she had mood swings and difficulty taking oral medication.

Her growth slowed during mitotane use from SDS + 1 (148.9 cm) to SDS-1 (158.5 cm). After discontinuation of mitotane she had a slow catch-up growth to SDS − 0.4 (166 cm). Her weight started above average due to Cushing syndrome (45.6 kg, SDS + 2 weight for height). During therapy, she maintained an above average weight for height, which returned to normal after discontinuation of treatment (55.8 kg, SDS + 0). Menarche was present at age 12 during mitotane therapy, after which her menses remained irregular, even after mitotane discontinuation. The adrenal function returned to normal one year after discontinuation of mitotane and all supportive medication were successfully discontinued.

A standardized neuropsychological assessment was performed three times during follow-up: at diagnosis (age 10), during mitotane treatment (age 12) and one year after mitotane discontinuation (age 14). Test scores were age-normalized and categorized into “below average”, “average” and “above average”. An impactful interruption of cognitive development occurred during mitotane therapy. At 12 years, her above average performance at school dropped to below average performance. Socially, it was difficult to keep up with classmates and friends, resulting in a social isolation. She needed to alter the type of high school, which impacted both herself and her family. Compared to the first measurement, she had the same verbal and performance intelligence, however, her test scores were limited by processing speed. Her processing speed and concentration had decreased significantly and fatigue during the test limited her performance. She complained of decreased short-term memory. However, when provided with enough time, no objective memory problems were found and memory was adequate for her school level. It was therefore concluded that her school performance and memory problems were secondary to decreased processing speed and concentration, worsened by fatigue and insecurity.

At age 14, after cessation of treatment, she recovered (nearly) completely, regained a higher school level and socially abilities were restored. Most importantly, her intelligence and short-term memory improved to above average. Her processing speed and concentration had improved, but remained slightly below average. Fatigue and insecurity were no longer an issue. It was concluded that she had shown a significant improvement on multiple cognitive levels.

## Review of the literature

We identified an additional 14 case reports and case series describing side-effects and neurotoxicity in 94 children with ACC or Cushing treated with EDP and/or mitotane (Table 1). Gastro-intestinal side-effects including nausea, vomiting, anorexia, requirement of a nasopharyngeal tube, diarrhea and abdominal pain were reported in nine studies [20, 22–28]. These side-effects were completely reversible after dose reduction or cessation of mitotane.

Mitotane was related to impaired development and endocrine function. Mitotane is known to cause adrenal insufficiency, requiring in most cases hydrocortisone and incidentally fludrocortisone supplementation. Two cases had an acute presentation of adrenal insufficiency and two cases had need for continued hydrocortisone supplementation after mitotane discontinuation [25, 27–29]. One case of central hypothyroidism was mentioned [25]. Mitotane was reported to have an effect on physical and hormonal development in pediatric patients resulting in growth impairment, increased bone age, gynecomastia, thelarche and central pubertal praecox [23, 25–27, 30, 31]. These effects were reversible after dose reduction or cessation of treatment in all but one case [25]. In this case, central pubertal praecox continued until one year after mitotane discontinuation for which leuproreline therapy was needed. The effect of endocrine activity of ACC itself also has an important effect on growth and development, especially the negative effects of cortisol production and the effects of sex hormones on pubertal development. However, it is generally observed that hormone levels return to normal after initial resection and adjuvant treatment [28, 29].

We found seven case reports and case series in children describing neurotoxicity of mitotane, with or without EDP. A case report from 2002 described a 2-month-old boy, who developed a motor and speech delay while treated with mitotane (levels > 10 mg/L). The neurological damage was reversible in this boy, with a normal intellect after 15 years, with only a slight weakness in organization and concentration [23]. Reversible speech delay was also found in four other reports [20, 24, 27, 32]. Two of these report side-effects with mitotane levels between 12 and 34.55 mg/L, the other two unfortunately only provided the mitotane dosage (1–2 and 4 g/day). In the case of Rashed et al., further diagnostic efforts into the speech delay let to the discovery of an arachnoid cyst with early hydrocephalic changes and no evidence for cerebral metastasis. The speech delay however, was more likely related to the mitotane, since improvement occurred after mitotane discontinuation [32]. Additionally reported neurotoxic side-effects were somnolence, lethargy, ataxia, asthenia, myalgia, vertigo and depression [12, 26–28]. Reported mitotane levels ranged from 10 to 29.8 mg/L. These effects were all reversible after mitotane dose reduction or discontinuation.

Two incidences of encephalopathy were described, both at toxic levels of mitotane (29.8 and 34.55 mg/L) [24, 27]. One of these cases presented with apathy, speech delay, memory disturbance, ataxia and mild quadriplegia. This patient fully recovered 6 months after mitotane discontinuation and had normal school performance three years after diagnosis [24]. The second patient developed high blood pressure followed by encephalopathy. This resolved after mitotane was temporarily discontinued. The patient made a complete recovery and resumed mitotane at a lower daily dosage [27].

Cognitive side-effects have also been reported at lower doses of mitotane. A study into the effect of mitotane as a treatment for juvenile Cushing Disease, found reduced school performance in two of the six patients. Both patients have fully recovered [28]. All of these case reports and case series suggest that neurotoxicity of mitotane is reversible in pediatric cases.

In addition to mitotane, EDP chemotherapy is also commonly applied in pediatric ACC. The side-effects of EDP are well described and include hearing loss, pancytopenia and neutropenic fever. Neuro-cognitive side-effects as a result of EPD have not been described for ACC or other pediatric solid tumor patients treated with similar combinations of chemotherapy [23, 26, 31].

### Comparison to side-effects and neurotoxicity in adults

In adults, similar side-effects and neurotoxicity have been described during EDP-mitotane, but differences exist [18, 33]. The most common side-effects of mitotane in adults are gastro-intestinal effects such as nausea, vomiting, diarrhea, constipation, flatulence, different taste and anorexia, similar to children. We have found two pediatric case of liver toxicity of mitotane, while in adults, this side-effect has been described more often [27, 28]. Endocrinological effects are also reported in both children and adults, such as adrenal insufficiency, hypogonadism, gynecomastia, irregular menses, ovary cysts and hypothyroidism [34]. While in children increased bone age has been described in two cases, in adults decreased bone mass has been reported [35]. However, this phenomenon in adults is mostly linked to cortisol excess, not to EDP-mitotane. Some known side-effects of mitotane have not (yet) been described in children, including skin rash and a high cholesterol. Side-effects related to EDP are comparable between adults and children, and include pancytopenia, hair loss, hearing loss, renal and liver toxicity [36].

Neurotoxicity of EDP-mitotane in adults includes somnolence, lethargy, ataxia, paresthesia, peripheral neuropathy, myasthenia, speech impairment (aphasia and dysarthria). Decreased coordination, vertigo and dizziness are also common. Cognitive impairment in adults usually presents with confusion, decreased concentration and memory impairment. Psychological effects have been reported with mood swings, panic attacks, burn-out and even clinical depression [37]. These neurotoxic effects appear to be partly or completely reversible after cessation of mitotane, same as in children [15, 38, 39]. In adults, the occurrence of neurotoxic and cognitive side-effects of mitotane, are described to be correlated to toxic plasma levels of > 20 mg/L [16]. Especially encephalopathy has been described only in children and adults with very high mitotane plasma levels (> 30 mg/l) [24, 27, 40]. Remarkably, our patient and most patients in the case reports had no plasma levels above the toxic limit and still had a significant impact on cognitive development during treatment. This warrants specific monitoring of cognitive development in children during treatment, even when plasma levels are within limits. However, there is insufficient data to justify a specific therapeutic target level for children based on the current review.

## Conclusions

EPD-mitotane therapy in children is associated with a range of side-effects including gastro-intestinal, developmental, endocrine and neuropsychological. Especially the gastro-intestinal and neuro-cognitive side-effects are attributable to mitotane and appear to be reversible after mitotane discontinuation. We have added our experience with a 10 year old girl who developed an impactful but reversible delay in cognitive development, measured with a standardized neuropsychological assessment multiple times during EDP-mitotane treatment. This case and the reports in the literature describe a similar course with sometimes severe, but reversible effects of EDP-mitotane, even in young children before the completion of normal development. This should provide reassurance to physicians, parents and patients in case EDP-mitotane treatment is indicated.

**Table 1:** Studies describing short- and long-term general side-effects and neurotoxicity in children treated with mitotane and/or EDP

Study	Population	Treatment	Mitotane dose/level	General side-effects	Neurotoxicity	Follow-up	Recovery
Case reports							
This report	10–year–old girl Stage IV ACCCushing and virilization	Resection, EDP–mitotane 2 years, metastasectomy	3 g/day 14–20 mg/L	Nausea, hypothyroidism, hypocalcemia, fluor vaginalis, irregular menses, growth impairment, fatigueEDP–related: neutropenia, anemia, mouth sores	Mood swings, memory impairment, reduced processing speed and concentration, decreased school performance	5 years	Disease free, normal school performance, mild concentration issues, irregular menses
Montgomery1965	3–year–old girl Stage IV ACCCushing	Mitotane	8 g/day	Nausea, vomiting			Good response, died from measles
Becker 1975	3.5–year–old boy Stage III ACCVirilization	Resection, mitotane 2.5 years	1 g/day	Gynecomastia, growth impairment, increased bone–age		4 years	Free of disease, growth recovery unknown
De León 2002	2–month–old boy Stage III ACC (rapid progression)Cushing	Resection, mitotane, re–resection	2.5 g/day >10 mg/L	Gynecomastia, anorexia, growth impairment	Motor and speech delay	15 years	Catch–up growth, average intellect, normal speech and motor function, weak organization and attention
Goto 2008	4–year–old boy Stage I ACCVirilization	Resection, mitotane 6 months, leuproreline	5 g/day 13.43–34.55 mg/L	Anorexia	Encephalopathy with apathy, speech and memory impairment, mild quadriplegia and ataxia	3 years	Complete recovery 6 months after mitotane discontinuation, disease free, normal school performance
Arai2008	10–year–old boy Stage IV ACCVirilization	Resection, EDcarboplatin–mitotane, metastasectomy	3 g/day	No severe side–effects		2 years	Disease free
Rashed 2017	3–year–old boy ALL + Stage II ACC (rapid progression)	Resection, re–resection, EDP–mitotane, metastasectomyALL: ALL–XV protocol; 6–MCP/MTX	4 g/day	Arachnoid cyst with hydrocephalus	Speech delay		Recovery of speech after mitotane discontinuation, died from progression of ACC
Oddie 2018	4–year–old girl Stage III ACCVirilization	Resection, EDP–mitotane	2.5 g/day	Gastro–intestinal, electrolyte abnormalities, febrile neutropenia, skin pigmentation, central pubertal praecox (gynecomastia, increased bone–age), central hypothyroidism		1 year	Continued hydrocortisone dependence, continued central pubertal praecox after mitotane discontinuation needing leuproreline
Takeuchi 2018	4.5–year–old boy Stage III ACCGynecomastia	Resection, EDP–mitotane (3 months presurgery), GPOH–MET97–mitotane^a^ (6 months postsurgery)		Unknown		2 years	Complete recovery of growth and gynecomastia, continued hydrocortisone/fludrocortisone dependence
Case series							
Ribeiro 2000	54 childrenMedian age 3 years Stage I–IV ACC	Resection, mitotane, EDP–mitotane		Nausea, vomiting, diarrhea, abdominal pain, gynecomastia/ thelarche	Somnolence, lethargy, ataxic gait, depression, vertigo	18 years	24 died, 30 disease free
Wajchenberg 2000	22 childrenAge 0–18 years Stage I–IV ACC22 virilization, 9 Cushing	Resection, 3 mitotane		Nausea, vomiting	Myalgia	5 years	3/3 on mitotane died19/22 free from disease
Zancanella 2006	11 children Age 2.5–15 years Stage III–IV11 virilization, 3 Cushing	11 EDP–mitotane, 7 resection, 5 metastasectomy	1.8–5.3 g/day 12–29.8 mg/L	Nausea, vomiting, 2/11 requiring nasogastric tube, diarrhea, abdominal pain, gynecomastia, 1/11 died from untreated acute adrenal insufficiencyEDP–related: pancytopenia (WHO1–4), hearing problems (WHO1), 1/11 renal clearance and liver function decrease (WHO1)	Speech delay, lethargy, ataxia, vertigo, 1/11 hypertensive encephalopathy	7.5 years	All side–effects were reversible between 1–3 weeks after mitotane discontinuation or dose reduction0.1/11 free from disease2/11 progression8/11 died from progression
Kostiainen 2019	4 children Age 4 months – 5 years Stage I–II1 Cushing, 2 virilization	EP		1/4 Central pubertal preacox requiring GnRH analogue		1.5–12 years	Disease free, normal growth and development
Zekri 2020	18 children Median age 4 years Stage I–IV ACC16 hormone production	14 resection (2 presurgery EDP), 8 EDP–mitotane	1–2 g/day 4g not tolerated	4/8 Nausea, vomiting, abdominal pain 1/8 Died from severe myelosuppression, cardiac toxicity, chest infection and sepsis	1/8 Speech delay		Recovery of speech after mitotane discontinuation8/8 stage III–IV died from progression10/10 stage I–II remain disease free
Motte 2018	6 children Mean age 12 years Juvenile Cushing disease	Mitotane >6 months	>10 mg/L	6/6 Nausea, vomiting2/6 Adrenal insufficiency (1/6 acute, 27 mg/L)1/6 Acute severe hepatitis (36 mg/L)	4/6 Asthenia2/6 Decreased school performance		Recovery after dose reduction or discontinuation

^a^GPOH-MET97 study [13]. *ACC* adrenocortical carcinoma, *ALL* acute lymphoid lymphoma, *EDP* etoposide, doxorubicin, cisplatin; *EP* etoposide, cisplatin, *6MCP/MTX* 6-mercaptopurine/methotrexate, *WHO* World Health Organization classification for adverse events

## Data Availability

Not applicable.
